# *Ophiocephalus striatus* Extract Supplementation Decreases Serum IL-6 Levels in Older People with Sarcopenia—A Single-Center Experience

**DOI:** 10.3390/geriatrics9020035

**Published:** 2024-03-07

**Authors:** Nur Riviati, Legiran Legiran, Irsan Saleh, Taufik Indrajaya, Zulkhair Ali, Irfannuddin, Probosuseno

**Affiliations:** 1 Internal Medicine Department, Medical Faculty, Sriwijaya University, Palembang 30126, Indonesia; 2 Biomedicine Department, Dr. Mohammad Hoesin Hospital, Faculty of Medicine, Sriwijaya University, Palembang 30139, Indonesia; dr.legiran@fk.unsri.ac.id; 3 Pharmacology Department, Dr. Mohammad Hoesin Hospital, Faculty of Medicine, Sriwijaya University, Palembang 30139, Indonesia; irsan_saleh_hasani@yahoo.com; 4 Internal Medicine Department, Dr. Mohammad Hoesin Hospital, Faculty of Medicine, Sriwijaya University, Palembang 30139, Indonesia; tfk_indrajaya@yahoo.com (T.I.); zulkhair@yahoo.com (Z.A.); 5 Physiology Department, Dr. Mohammad Hoesin Hospital, Faculty of Medicine, Sriwijaya University, Palembang 30139, Indonesia; irfan.med@unsri.ac.id; 6 Internal Medicine Department, Faculty of Medicine, Gajah Mada University, Yogyakarta 55281, Indonesia; probosuseno@ugm.ac.id

**Keywords:** sarcopenia, *Ophiocephalus striatus*, IGF-1, IL-6, older adults

## Abstract

Sarcopenia, a condition characterized by muscle loss and decreased function in older adults, is a growing public health concern. This study aimed to investigate the effects of *Ophiocephalus striatus* extract on insulin-like growth factor-1 serum, interleukin-6 serum levels, and sarcopenia-related parameters in older adults with sarcopenia. This double-blind randomized controlled trial included 80 older adults with sarcopenia. Participants were randomly assigned to receive *Ophiocephalus striatus* extract or a placebo for two weeks. The IGF-1 serum and IL-6 serum levels were assessed as primary outcomes. The *Ophiocephalus striatus* extract intervention resulted in a significant reduction in serum IL-6 levels. Although the IGF-1 levels did not show significant changes, there was an increase for the intervention group. This study demonstrated that a 2-week intervention with Ophiocephalus striatus extract positively impacted the serum IL-6 levels in older adults with sarcopenia. While the IGF-1 levels did not change significantly in this short intervention period, the observed improvements in IGF-1, calf circumference, muscle mass, and muscle strength are promising. The findings suggest that Ophiocephalus striatus extract may offer a valuable intervention for managing sarcopenia, particularly in regions with abundant Ophiocephalus striatus production, such as South Sumatera. This study was registered with trial number NCT05869383.

## 1. Introduction

The increasing elderly population is accompanied by age-related health problems. One such problem is the widespread and progressive loss of muscle mass and strength known as sarcopenia. The diagnosis of sarcopenia is based on the Asian Working Group for Sarcopenia (AWGS) criteria, which involve screening with SARC-F or calf circumference and assessing muscle mass, strength, and physical performance [1]. Sarcopenia is now recognized as a muscle disease in the International Classification of Diseases, Tenth Revision (ICD-10) code M62.84 issued by the World Health Organization (WHO) in 2016 [2]. Sarcopenia has serious implications for the elderly, including falls, fractures, hospitalizations, a decreased functional status, a reduced quality of life, an increased risk of postoperative infection, susceptibility to nosocomial infections, community-acquired pneumonia, 90-day mortality in patients with aspiration pneumonia, disability, frailty, morbidity, and mortality [2,3,4,5,6,7].

One of the main factors contributing to sarcopenia is low-grade systemic inflammation and disrupted muscle protein homeostasis. Inflammation in the elderly can occur due to changes in body structure, including increased adipose tissue that secretes interleukin 6 (IL-6) and Tumor Necrosis Factor-α (TNF-α), leading to chronic low-grade inflammation known as aging inflammation [8]. With advancing age, cytokine production increases, resulting in elevated IL-6 levels, which increases the risk of sarcopenia [8]. Studies have shown a correlation between IL-6 levels and the severity of sarcopenia, age, and comorbidities in the elderly [9]. Chronic low-grade inflammation can activate catabolic pathways, leading to protein breakdown and the inhibition of protein synthesis, ultimately resulting in muscle loss [9]. The age-related loss of skeletal muscle mass is also caused by decreased protein synthesis, which is regulated by mTOR1 through intracellular signaling [10]. One of the factors that can induce mTOR-1 is insulin-like growth factor-1 (IGF-1). IGF-1 is a potent hormone that stimulates protein synthesis and inhibits protein catabolism [10]. It plays a role in the growth, differentiation, and regeneration of skeletal muscle and has the potential to be a therapeutic factor in reducing atrophy and frailty in sarcopenia [10]. Low levels of IGF-1 have been found in elderly patients with sarcopenia compared to those without sarcopenia. There is an independent association between IGF-1 and muscle mass, and low IGF-1 levels are associated with reduced muscle strength and limited physical performance [11,12,13]. A low protein intake reduces plasma IGF-1 levels, while an increased protein intake can raise IGF-1 levels. Elevated IGF-1 levels enhance protein synthesis and inhibit protein degradation [14].

Comprehensive management recommendations for sarcopenia include non-pharmacological approaches such as resistance training and nutrition [15,16]. Adequate nutrition, including protein supplementation, is a modifiable factor and the most widely recommended strategy used to address sarcopenia [15,16]. Systematic reviews and meta-analyses have shown an association between inadequate protein intake and sarcopenia in the elderly. However, a clinical trial on sarcopenia using a whey protein product with a dose of 6 g/day did not show significant effects on the IL-6 and TNF-α levels [17].

*Ophiocephalus striatus*, also known as snakehead or gabus fish, is a freshwater fish in South Sumatra, Indonesia. It is a popular local delicacy and a rich source of protein with essential and non-essential amino acids, essential polyunsaturated fatty acids (PUFA) omega-3, albumin, minerals, zinc, and vitamins [12,18,19,20,21]. Scientific studies have demonstrated the beneficial effects of *Ophiocephalus striatus* in increasing albumin levels, which has anti-inflammatory and cell proliferation properties. It can also increase IGF-1 levels and decrease IL-6 levels, which are important factors in sarcopenia. Several studies have shown decreases in IL-6 and TNF-α levels after using *Ophiocephalus striatus* extract [12,18,22]. A study on cancer patients with cachexia found a significant reduction in IL-6 levels after two weeks of using *Ophiocephalus striatus* extract [18]. Another study on elderly patients with malnutrition showed an increase in IGF-1 levels after two weeks of *Ophiocephalus striatus* extract administration [12]. Assessing IL-6 and IGF-1 as the primary biochemical markers of sarcopenia was driven by their accessibility in terms of measurement in Palembang. IGF-1 was selected to represent anabolic markers, reflecting the body’s capacity for muscle tissue synthesis, while IL-6 was utilized as a marker for inflammation, which can disrupt anabolic processes and promote catabolism. These mechanisms are recognized as pivotal in the pathogenesis of sarcopenia, particularly in aging individuals. Although the significances of additional markers such as irisin, tumor necrosis factor, and interleukin-8 in understanding the multifaceted nature of sarcopenia are acknowledged, their measurements in our study were precluded by logistical constraints and resource limitations [23]. Nonetheless, valuable insights into the complex interplay of factors contributing to the development and progression of sarcopenia could be gained from future investigations incorporating these markers. Based on the information mentioned above and the lack of research on the effects of *Ophiocephalus striatus* on sarcopenia, this study aimed to investigate the effects of *Ophiocephalus striatus* extract on the IGF-1 and IL-6 levels in older adults with sarcopenia. The results of this study are expected to provide valuable insights for clinicians in managing elderly patients with sarcopenia.

## 2. Materials and Methods

This study was a double-blind randomized controlled trial aimed at determining the effects of *Ophiocephalus striatus* extract on the IGF-1 and IL-6 levels in older adults with sarcopenia. The study was conducted at the Geriatric Clinic of RSUP Dr. Mohammad Hoesin, Palembang. Ethical clearance was obtained from the Research Ethics Commission (No. DP.04.03/D.XVIII.6.11/ETIK/36/2023). This study spanned from March 2023 to August 2023. The target population consisted of elderly patients aged 60 years and above with sarcopenia who sought treatment at the Geriatric Clinic of RSUP Dr. Mohammad Hoesin, Palembang. This RCT was registered under trial registration number NCT05869383.

### 2.1. Inclusion Criteria

Older adults (≥60 years old, in line with the Indonesian Ministry of Health Regulation) with sarcopenia were diagnosed using AWGS criteria, which include low muscle mass (estimated appendicular skeletal muscle index BIA cut-off of <7.0 kg/m^2^ for males, <5.7 kg/m^2^ for females), low muscle strength (measured using a handgrip dynamometer: <28 kg for males, <18 kg for females), and low physical performance (assessed using the 6 m walk test: <1 m/s).

### 2.2. Exclusion Criteria

Patients with severe chronic liver disease or elevated SGPT levels > 3 times the upper limit of normal, impaired kidney function with an estimated glomerular filtration rate < 30 mL/min without hemodialysis, acute disease phase (e.g., acute infection, acute arthritis, acute stroke, trauma), malignancy, depression according to the Geriatric Depression Scale (score > 10), history of hypersensitivity to *Ophiocephalus striatus*, or that refused to participate in the study were excluded from this study.

### 2.3. Drop-Out Criteria

Subjects were considered dropped if subjects died before day 14, were lost to follow-up, experienced severe adverse effects, developed acute conditions during the follow-up period, or withdrew from participation before the completion of the study.

### 2.4. Data Collection

The data collection included sociodemographic information, comorbidities based on patient history and medical records, physical examinations, anthropometric measurements (height, weight, upper arm and calf circumferences), nutrition assessment (using the Mini Nutritional Assessment and 3-day food records), a functional status assessment (using the Barthel Activities of Daily Living score), mental status assessment (using the Geriatric Depression Scale), cognitive status assessment (using the Abbreviated Mental Test), sarcopenia screening (using the SARC-F questionnaire and calf circumference), a handgrip strength assessment (using the Jamar Hydraulic Hand Dynamometer Model J00105), a 6 m walk speed assessment, a muscle mass assessment (using Bio Impedance Analysis TANITA BC-545N), and laboratory tests for IGF-1 and IL-6. The selected BIA model was chosen due to its widespread use in clinical settings across Indonesia, and the BIA is supported by current guidelines, while the DXA is not available in Palembang, and Indonesia has only one, located in Jakarta.

In this study, the type of sarcopenia under investigation was not limited. Instead, subjects who met the criteria for sarcopenia as defined by the Asian Working Group for Sarcopenia (AWGS) in 2019 and who also fulfilled all the inclusion criteria specified for the study were included. By adhering to these inclusion criteria and utilizing the AWGS guidelines for sarcopenia diagnosis, a standardized approach for participant selection was ensured, allowing sarcopenia to be investigated comprehensively across all its severity levels as outlined by the AWGS criteria. Consequently, a diverse range of individuals with sarcopenia was encompassed in the study, facilitating a thorough examination of the condition without restricting it to specific subtypes or severity levels.

### 2.5. Intervention

In this clinical trial, older adults visiting the Geriatric and Internal Medicine Clinic of RSUP Dr. Mohammad Hoesin, Palembang, were consecutively selected to participate. Once eligible subjects were identified, they were asked to participate in the clinical trial by providing informed consent and agreeing to undergo various assessments. Consent was obtained from the family for patients with cognitive impairment. Baseline data were collected before starting the intervention.

The eligible subjects were randomly allocated into two groups: one receiving 5 g of *Ophiocephalus striatus* extract twice a day and the other receiving a placebo for 2 weeks. The placebo contained maltodextrin, 5 g. The research subjects took supplements every morning and evening after meals. Both groups received a diet tailored to their individual needs based on comorbidities assessed by the dietitian. Randomization was performed using the permuted block randomization technique. The intervention codes were concealed in sealed envelopes to maintain blindness. The type of supplement administered (*Ophiocephalus striatus* extract or placebo) was known only to a third party. The main researcher, research assistant, attending physician, and research subjects were unaware of the type of therapy provided, ensuring a double-blind study design. Participants were allowed to continue their usual lifestyle, habits, physical activity, exercise, and other medications during the study.

To ensure compliance with the supplement consumption, the supplements were provided in sealed plastic packaging, which participants were instructed to open before consumption. The number of supplements consumed was recorded during the follow-up visit at the end of the second week after randomization. Participants were also provided with a diary to record their daily food and beverage intake. Any significant events, such as hospitalization or death, were reported to the researcher through telephone. Participants were encouraged to contact the researcher if they experienced any complaints or side effects. If feasible, participants were asked for their consent to continue supplement consumption.

At the end of the second week after randomization, participants underwent a follow-up visit where data on medical history, physical examination, anthropometric measurements, nutrition assessment, and laboratory tests for IGF-1 and IL-6 were collected. Participants who completed the entire prescribed supplement dose until the end of the second week were considered to have completed the study.

### 2.6. Outcome Measures

The primary outcomes of this study were the serum levels of IGF-1 and IL-6. IGF-1, a critical marker of growth and anabolic status, served as a primary indicator of the intervention’s potential to stimulate muscle growth and regeneration. IL-6, a primary inflammatory marker, was used to assess the impact of the intervention on reducing inflammation, which is often associated with muscle health and sarcopenia. IGF-1 and IL-6 were measured using Cloud-Clone Corp^®^ ELISA reagent with serial numbers 6854322BA3 and 41148D2B69, respectively.

In addition to the primary outcomes, several secondary outcomes were measured to provide a comprehensive evaluation of the intervention’s effectiveness. Calf circumference, a key screening parameter following the AWGS 2019 criteria, was included as a measure of muscle mass and overall muscle health. The measurement of the appendicular skeletal muscle mass index (ASMI) allowed us to assess changes in muscle mass among both male and female participants. This parameter is particularly relevant in evaluating the impact of the intervention on sarcopenia. Handgrip strength, a reliable indicator of upper extremity muscle function and overall muscle strength, was another secondary outcome assessed in this study. Furthermore, gait speed, an essential parameter for evaluating physical performance and mobility, was assessed among the participants.

### 2.7. Data Processing and Analysis

The collected data were recorded on research forms and subsequently transferred to electronic storage media for data cleaning and coding. Descriptive data and analysis are presented in narrative/text form or tables or graphics. Numeric data are presented as the means with standard deviations for normally distributed data or medians with interquartile ranges for non-normally distributed data. Categorical data are presented as percentages. The data analysis was performed using SPSS 21. A statistical hypothesis test was conducted using paired *t*-tests (intragroup effect) and independent *t*-tests (intergroup effect) for normally distributed data, and the Wilcoxon test (intragroup effect) and Mann–Whitney U tests (intergroup effect) were conducted for non-normally distributed data to assess differences in measured variables. A *p*-value < 0.05 was considered statistically significant.

## 3. Results

**Figure 1:**
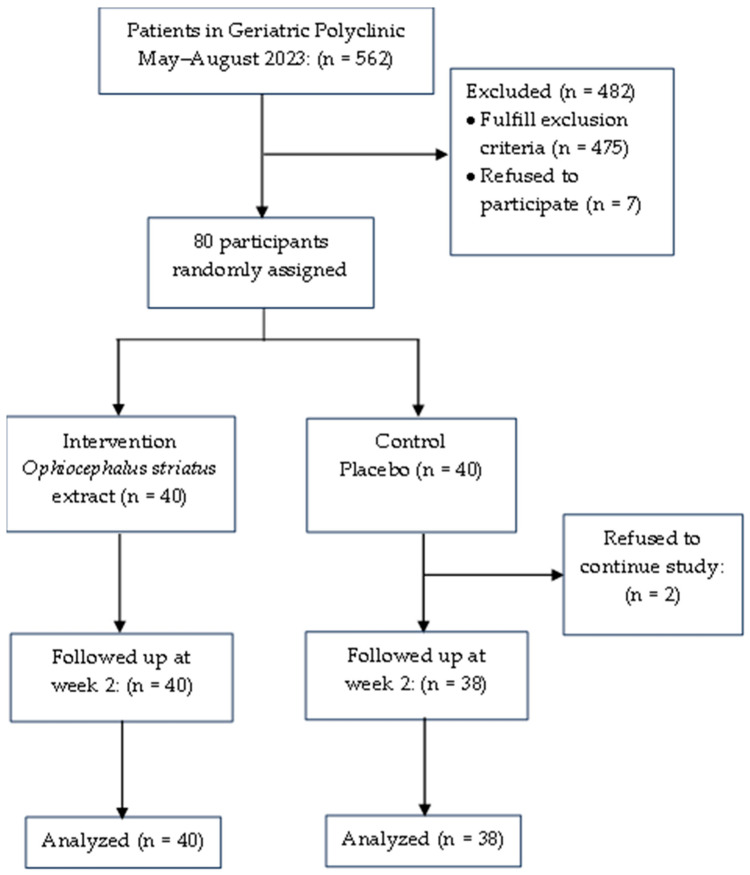
Consolidated Standards of Reporting Trials (CONSORT) diagram.

A total of 562 older adults from the Geriatric Polyclinic of Dr. Mohammad Hoesin Hospital were screened for sarcopenia based on their calf circumference, in line with the AWGS 2019 algorithm for screening sarcopenia. We excluded 482 patients because they met the exclusion criteria. Finally, this study included 80 elderly participants diagnosed with sarcopenia based on rigorous exclusion criteria. The Consolidated Standards of Reporting Trials (CONSORT) diagram is shown in Figure 1.

The prevalence data revealed that 14.23% of the elderly population in our study exhibited sarcopenia with an average age of approximately 70 years. A sex-based analysis in our study indicated a prevalence of 15.1% in males and 13.7% in females. Overall, the patient characteristics between both groups are relatively the same, except for their daily calorie, carbohydrate, and zinc intakes, which the placebo group had higher intakes than the intervention group. The patient’s characteristics are shown in Table 1.

After 14 days of intervention, we observed significant changes in several important indicators. Calf circumference showed a significant mean increase of 0.53 cm in the intervention group, while in the placebo group, there were no significant changes. The interclass correlation coefficient (ICC) for the calf circumference measurement was 0.85. Further analyses revealed that the significant increase was solely evident among women participants in the intervention group, with no significant increase observed among men participants in the same group. However, there was no significant treatment effect for calf circumference between the intervention group and the placebo group (*p*-value = 0.16). Insulin-like growth factor-1 (IGF-1) levels increased in the intervention group, but the difference was not statistically significant, with a mean change of 5.32 ng/dL in both groups. However, interleukin-6 (IL-6) levels demonstrated a remarkable decrease in the intervention group, both in men and women, with an overall mean reduction of 36 pg/dL, while the placebo group exhibited a substantial increase of 24.92 pg/dL.

In the intervention group, the appendicular skeletal muscle mass index (ASMI) increased in both men and women, although a significant increase was found only in women. The result does not show significant mean differences between the intervention and placebo groups, with mean differences of −0.08 kg/m^2^ and 0.04 kg/m^2^, respectively. Handgrip strength improved in both sexes, with a significant increase of 0.87 kg in women in the intervention group. Gait speed also showed improvements, with a mean increase of 0.21 m/s in men in the intervention group. Creatinine levels remained largely unchanged, with a mean difference of 0.01 between the intervention and placebo groups. The outcomes measured after 2 weeks of intervention are shown in Table 2.

## 4. Discussion

### 4.1. Prevalence of Sarcopenia by Age and Sex

In this study, the prevalence of sarcopenia was found to be 14.23% among 80 elderly individuals after screening and exclusion criteria from a total of 562 elderly individuals at RSUP DR. Mohammad Hoesin, Palembang between May and September 2023. This prevalence falls within the range of sarcopenia prevalence reported in the systematic review and meta-analysis conducted by Petermann-Rocha et al. (2022), which ranged from 10% to 27% when using EWGSOP criteria [24]. It also aligns with the prevalence range based on epidemiological studies using AWGS criteria, as reported by Chen et al. (2020), which varied from 5.5% to 25.7% among an entire elderly population [1].

The mean age of the intervention group was 70.28 ± 5.65 years, while the control group had a mean age of 71.34 ± 7.99 years. The median ages for the intervention and control groups were 70 years (range: 62–85 years) and 71 years (range: 60–88 years), respectively. Globally, the reported prevalence of sarcopenia is approximately 5–13% in individuals aged 60 to 70, increasing to 11–50% in individuals over 80 years old [25]. The aging process is often accompanied by a gradual replacement of muscle fibers with fibrotic or fatty tissue, exacerbated by oxidative damage and decreased antioxidant function. These age-related changes contribute to the higher risk of developing sarcopenia in older adults [26].

Out of the 205 elderly participants, 31 were male, and among the 357 female participants, 49 had been studied. This indicates that the prevalence of sarcopenia was 15.1% for males and 13.7% for females in this study. These findings are consistent with a systematic review and meta-analysis by Shafiee et al. (2017), reporting a sarcopenia prevalence of 13% (range: 7–19%) in males and 13% (range: 9–19%) in females. Other studies also note that in Asia, the prevalence of sarcopenia ranges from 5.1% to 21% in males and from 4.1% to 16.3% in females [1]. Similar results were also identified in the systematic review and meta-analysis by Petermann-Rocha et al. (2022), indicating that sarcopenia prevalence was higher in males when using EWGSOP criteria but higher in females when using International Working Group of Sarcopenia criteria [24].

### 4.2. Effect of Ophiocephalus Striatus Extract on Calf Circumference

After two weeks of intervention, a significant increase in calf circumference was observed in the intervention group, reaching approximately 27.76 cm (*p*-value = 0.019), specifically in women (*p*-value = 0.02). The variance in physical activity levels between sexes could potentially account for this distinction. However, it is noteworthy that there was no significant treatment effect observed in calf circumference between the intervention and placebo groups, which could be attributed to the relatively short duration of the intervention and the lack of exercise stimulation required to promote muscle growth. Calf circumference is not only important for diagnosing sarcopenia, but also useful in assessing muscle abnormalities in various medical conditions [27,28].

### 4.3. Effect of Ophiocephalus Striatus Extract on Serum IGF-1

This study showed an increase in the average IGF-1 levels in the intervention group, both in men and women; although it was not statistically significant, while no increase was observed in the control group. This lack of significance could be due to the relatively short duration of the treatment. IGF-1 plays a crucial role in muscle strength and development. Preclinical experiments have demonstrated that IGF-1 is associated with muscle mass and strength development [29]. IGF-1 reduces muscle degeneration and prevents excessive inflammation caused by toxins [29,30]. Additionally, IGF-1 increases the proliferation capacity of muscle satellite cells, which are essential for muscle repair and growth [29,30]. 

### 4.4. Effect of Ophiocephalus Striatus Extract on Serum IL-6

In this study, the baseline levels of IL-6 in the intervention group were higher (44.51 ± 23.57 pg/mL) than in the control group (36.47 ± 17.45 pg/mL). These results align with the findings of Rong et al. (2018), which reported lower IL-6 levels in the sarcopenia group compared to the non-sarcopenia group, indicating a significant association between IL-6 levels and muscle mass [23]. Erfin et al. (2021) also found that increasing IL-6 levels are positively correlated with decreased muscle mass, muscle strength, and physical activity. The lower baseline IL-6 levels in the control group can be attributed to their higher protein intake and physical activity levels.

While there have been no studies specifically investigating the impact of *Ophiocephalus striatus* extract on IL-6, several clinical trials have examined the effectiveness of fish oil intervention on IL-6. Muldoon et al. (2015) found no significant changes in IL-6 levels between a fish oil intervention group and a soybean oil control group [31]. In contrast, Ramirez et al. (2013) observed a significant decrease in IL-6 levels in an intervention group given 4 g/day of fish oil compared to the placebo group over 12 months in patients with multiple sclerosis [32]. Another study, by Janice et al. (2012), reported a 12% decrease in IL-6 in a high-dose fish oil intervention group compared to a 36% increase in IL-6 in the placebo group over a 4-month treatment period [33]. These studies collectively suggest that fish oil may have varying effects on IL-6 levels in different populations, possibly due to differences in dosage and the duration of intervention.

The changes in body composition during aging, such as the expansion of adipose tissue, especially visceral fat, can lead to the recruitment of monocytes and the polarization of monocytes toward inflammatory cells, including IL-6. IL-6 is a multifunctional cytokine with both pro-inflammatory and anti-inflammatory roles, with its effects depending on the target structure, dominant cytokine environment, and mode of release. In the context of aging inflammation, prolonged exposure to IL-6 signaling is involved in the pathogenesis of sarcopenia. Low IL-6 levels can stimulate satellite cell activity and myotube regeneration, while chronic IL-6 production can trigger muscle wasting. Increased levels of IL-6 are associated with the accumulation of intramuscular fat tissue, which significantly influences muscle function.

The positive impact of the *Ophiocephalus striatus* extract treatment on IL-6 serum levels in older adults with sarcopenia is possible because of its high protein and albumin content. *Ophiocephalus striatus*, commonly known as snakehead fish, is notably rich in essential amino acids, particularly albumin, which has been associated with immune system enhancement that can lead to IL-6 reduction [34]. Previous studies have highlighted the role of protein intake in inhibiting IL-6 expression, indicating a possible correlation between protein consumption and IL-6 regulation [35]. Moreover, snakehead fish contains high levels of protein and albumin, which are vital for reducing inflammation [36]. Additionally, its protein composition suggests a possible ability to regulate inflammatory markers like IL-6 [37]. Furthermore, protein supplementation has been linked to the restoration of glutathione levels, which play a crucial role in mitigating oxidative stress and inflammation [38]. The anabolic role of dietary protein has been associated with a decrease in the biomarkers of inflammation, including IL-6, particularly in older individuals [39]. This potential mechanism could explain IL-6 reduction following *Ophiocephalus striatus* extract supplementation in older adults with sarcopenia.

### 4.5. Effect of Ophiocephalus Striatus Extract on ASMI

In this study, the average ASMI for men before treatment was approximately 6.49 kg/m^2^ for the intervention group and 6.42 kg/m^2^ for the control group. For female patients, the average ASMI was around 5.42 kg/m^2^ for the intervention group and 5.48 kg/m^2^ for the control group. The average ASMI increased from approximately 6.49 kg/m^2^ to 6.56 kg/m^2^ after treatment, although this change was not statistically significant (*p*-value = 0.362). For women, the average ASMI increased from around 5.43 kg/m^2^ to 5.58 kg/m^2^, and this change was statistically significant (*p*-value = 0.039).

The results presented by Zhu et al. (2015) for postmenopausal older women, involving 93 participants in an intervention group who received high-protein milk supplements (30 g protein) and 88 participants in the placebo group who received isocaloric milk (2.1 g protein) revealed no significant association between muscle mass and other physical functions over a 2-year treatment period. This study suggests that protein supplementation may be more effective in older adult populations with a previously low protein intake [40]. Moreover, protein intake should ideally be combined with muscle training to stimulate concurrent muscle protein [41].

Consuming higher protein levels than currently recommended (RDA, 0.8 g/kg/day) can be used as a strategy to maintain muscle mass and physical function at older ages. This recommendation is based on the fact that aging muscles require more amino acids to maximally stimulate muscle protein synthesis in response to hyperaminoacidemia, a phenomenon known as anabolic resistance. Sufficient protein consumption is expected to increase amino acid availability and stimulate sarcoplasmic and myofibrillar muscle protein synthesis. Failure to stimulate muscle protein synthesis through nutrition predisposes gradual muscle loss, particularly of type II muscle fibers, impacting muscle strength and physical function [42]. The precise total protein dosage needed for older adults to gain muscle mass and muscle strength still varies, and clear guidelines are yet to be established.

### 4.6. Effect of Ophiocephalus Striatus Extract on Muscle Strength

In this study, an increase in handgrip strength was observed in the group that received *Ophiocephalus striatus* 2 × 5 g, especially in women, although this increase was not statistically significant in men. This difference may be influenced by varying levels of physical activity between men and women. These results indicate that physical exercise may play a more crucial role in improving muscle strength compared to protein supplementation. *Ophiocephalus striatus* is rich in protein, which is essential for muscle protein synthesis, and branched-chain amino acids (BCAA) that play a vital role in promoting the growth of skeletal muscle. Additionally, the high albumin content in *Ophiocephalus striatus* has antioxidant effects that can help protect muscles from oxidative damage. The use of *Ophiocephalus striatus* as a dietary supplement to enhance muscle strength requires further research to explore its potential effectiveness.

### 4.7. Effect of Ophiocephalus Striatus Extract on Gait Speed

In this study, a significant increase in walking speed was observed in the overall population following *Ophiocephalus striatus* supplementation. However, the impact of amino acid interventions on walking speed remains a topic of ongoing investigation, with varying results in the existing literature. For instance, clinical trials involving β-hydroxy β-methylbutyrate (HP-HMB) and other amino acids have often yielded non-significant improvements in walking speed [42]. These discrepancies in findings may be due to differences in amino acids, dosages, treatment durations, and participant characteristics. Walking speed is influenced by various factors beyond muscle mass and strength, making it a multifaceted parameter [43].

### 4.8. Effect of Sarcopenia Severity on Intervention Response

Sarcopenia, especially in its severe form, represents an advanced stage of muscle loss and decreased functional capacity compared to common sarcopenia. This advanced stage is often associated with more significant impairments in physical performance and an increased risk of frailty [44]. Individuals with severe sarcopenia may experience a more pronounced disruption in muscle protein turnover and homeostasis, necessitating more intensive interventions to enhance muscle mass and strength.

Protein supplementation has been investigated as a potential intervention for severe sarcopenia and sarcopenia. Protein is vital for muscle protein synthesis and plays a crucial role in maintaining and building muscle mass. Studies have shown that protein supplementation, when combined with exercise therapy, can lead to increased muscle mass, strength, and physical performance in older adults with sarcopenia [44]. However, the response to protein supplementation may differ between severe and common sarcopenia. Individuals with severe sarcopenia may experience more severe muscle loss and functional decline, necessitating a more intensive approach to protein supplementation. Higher protein doses or longer supplementation durations may be required to achieve a significant increase in muscle mass and strength [44].

Conversely, individuals with common sarcopenia may respond positively to lower doses of protein supplementation. Studies have shown that even slight increases in protein intake can lead to improved muscle mass and strength in this population [45]. However, the optimal dosage and duration of protein supplementation for managing sarcopenia may vary depending on individual factors such as age, sex, and overall health status. 

### 4.9. Influence of Nutritional Intake

In this study, differences in nutritional intake were observed between the intervention and control groups. Overall, the control group had a higher daily calorie intake compared to the intervention group. Additionally, the intervention group had a slightly lower protein intake compared to the control group, although this difference was not statistically significant. In terms of micronutrients, the control group had a higher daily zinc intake compared to the intervention group. These disparities could potentially affect the response to the treatment in this study.

Understanding the impact of total calorie intake on muscle strength in older adults is a complex relationship that involves various factors. Caloric intake provides the energy required for muscle contractions and overall physical activity. Previous research has found that inadequate calorie, protein, and leucine intakes are associated with reduced muscle mass in older adults who have experienced hip fractures [46]. This suggests that an insufficient calorie intake can contribute to muscle mass loss in older adults. Conversely, calorie restriction may have a negative effect on muscle strength. Other studies have indicated that calorie restriction leads to increased muscle mitochondrial biogenesis in healthy humans [47]. However, it should be noted that prolonged calorie restriction without adequate protein intake can lead to muscle damage and decreased muscle strength.

In addition to calorie intake, protein intake plays a crucial role in influencing muscle strength in older adults. Protein supplementation has shown potential in increasing muscle mass in older adults with sarcopenia. Studies have demonstrated that older women who exercise and consume additional amino acids rich in leucine experience increased leg muscle mass and muscle strength and a faster walking speed [48]. Leucine is an essential amino acid that plays a key role in stimulating muscle protein synthesis [49]. Other research has found that leucine or leucine-enriched protein significantly improved sarcopenia in older individuals by increasing muscle mass content [49]. Observational data also suggest that a higher protein intake is associated with increased muscle mass and better muscle function in older adults [50]. A systematic review and meta-analysis found that milk protein supplementation, especially whey protein, can increase lean body mass and muscle strength in older adults [51]. Another systematic review and meta-analysis found that protein intake from dairy products is associated with increased muscle mass, muscle strength, and physical performance in older adults, whether they have sarcopenia or not [52]. Consuming an adequate amount of protein is crucial for maintaining and enhancing muscle protein synthesis, which is required for muscle growth and maintenance.

Zinc is a crucial mineral that plays a pivotal role in various physiological processes, including protein synthesis, immune function, and cell growth [53]. Protein synthesis is a fundamental process for muscle growth and repair. Therefore, zinc, as an additional factor in protein synthesis, can indirectly impact muscle strength. Moreover, some references discuss the role of zinc in regulating specific proteins and signaling pathways related to muscle strength. Zinc regulates the activities of certain proteins and signaling pathways, such as AMP deaminase and the Akt pathway, which are associated with muscle strength. Furthermore, various factors related to muscle strength, such as muscle metabolism and muscle fibers, may be influenced by zinc intake [53,54]. Zinc is known to interact with various enzymes and proteins involved in muscle metabolism and muscle function [54]. Therefore, zinc intake can indirectly affect muscle strength by influencing the regulation of these proteins and signaling pathways.

### 4.10. Influence of Physical Activity Level on Intervention Response

Physical activity’s role in sarcopenia management is essential, with guidelines recommending a combination of nutritional intervention and exercise for older adults [1,27]. Although this study found no statistically significant difference in the physical activity levels between the intervention and control groups, it is important to note that the control group had higher activity levels than the intervention group. Exercise, especially resistance training, offers several benefits for sarcopenia management [55]. It stimulates muscle protein synthesis, boosts anabolic hormones, activates satellite cells, enhances mitochondrial function and and neural adaptations, and imposes mechanical stress on muscles [51,56,57].

### 4.11. Tolerability and Safety

This study analyzed intervention-related side effects and found that they were generally mild and non-disruptive, indicating that *Ophiocephalus striatus* supplementation was well-tolerated by most patients [58,59]. Importantly, the intervention did not significantly affect kidney function, aligning with prior research findings. Taking *Ophiocephalus striatus* after meals or considering proton pump inhibitors may help mitigate gastric-related side effects [60].

### 4.12. Clinical Relevance

This pioneering study on *Ophiocephalus striatus* extract’s impact on sarcopenia yields significant clinical implications. Notably, the intervention significantly reduces IL-6 levels, offering prospects for managing chronic inflammation and related diseases. Although the rise in insulin-like growth factor 1 (IGF-1) is not statistically significant, it hints at the extract’s potential to affect growth hormone status in sarcopenic patients. Notably, calf circumference, muscle mass, grip strength, and walking speed significantly improved in the intervention group over two weeks. This boost in functionality is pivotal for improving the quality of life in sarcopenic patients.

The study’s short duration and affordable dose underscore its clinical relevance, particularly since *Ophiocephalus striatus* is abundant in South Sumatra, suggesting regular consumption for improved sarcopenia management. This offers a valuable option for community-level intervention, with collaboration among government bodies, healthcare practitioners, and producers in high-production regions playing significant roles in advocating for this approach.

Considerable limitations impacted the result interpretation. Differences in nutritional intake, zinc intake, and physical activity levels between groups, although not statistically significant, affected the baseline conditions and patient response. Separating groups into sarcopenia and severe sarcopenia may mitigate potential bias. The selected BIA model had a limitation concerning the absence of bibliographic references for the appendicular skeletal muscle (ASM) regression equation specific to this model as well as of a validation study to use against a reference method. Conducting research across diverse regions is advisable for broader applicability. Exploring optimal dosages and potential synergies with exercise in sarcopenia management can strengthen this study’s findings. This in-depth research can enhance our understanding of *Ophiocephalus striatus*’s effects on sarcopenia and inform management in the older adult population.

## 5. Conclusions

In conclusion, this study demonstrates that a 2-week intervention with *Ophiocephalus striatus* extracts reduces serum IL-6 levels in older adults with sarcopenia. While limitations concerning the physical activity and nutrition intake existed, the safety profile of the intervention is promising. Further investigations are needed to explore the effects of *Ophiocephalus striatus* extract to establish optimal dosages and their synergy with other treatment modalities such as exercise. These findings hold significant clinical relevance, particularly in regions with a high *Ophiocephalus striatus* production like South Sumatera, suggesting the potential for improved sarcopenia management through the regular consumption of this natural resource.

## Figures and Tables

**Table 1:** Baseline characteristics of subjects.

Characteristics	Intervention *Ophiocephalus striatus *(n = 40)	Control Placebo (n = 38)	*p*-Value
Age, median (min–max)	70 (62–85)	71 (60–88)	0.627
60–65 years	9 (22.5)	11 (28.9)	0.137
66–70 years	12 (30)	5 (13.2)	
71–75 years	14 (35)	10 (26.3)	
≥76 years	5 (12.5)	12 (31.6)	
Sex, n (%)			0.854
Men	15 (37.5)	16 (42.1)	
Women	25 (62.5)	22 (57.9)	
Smoking status, n (%)			0.431
Yes	16 (40)	11 (28.9)	
No	24 (60)	27 (71.1)	
Nutrition status, n (%)			0.799
Normal	7 (17.5)	7 (18.4)	
At risk for malnutrition	28 (70)	28 (73.7)	
Malnutrition	5 (12.5)	3 (7.9)	
ASMI (kg/m^2^), median (min–max)			
Men	6.6 (5.86–6.90)	6.57 (4.92–6.80)	0.565
Women	5.60 (4.70–5.68)	5.54 (5.00–5.68)	0.716
Handgrip strength (kg), mean ± SD			
Men	17.27 ± 5.26	21.38 ± 6.35	0.060
Women	17.48 ± 6.15	19.55 ± 6.86	0.282
Gait speed (m/s), mean ± SD	0.70 ± 0.20	0.69 ± 0.22	0.886
Sarcopenia severity			0.662
Sarcopenia	17 (42.5)	19 (50)	
Severe sarcopenia	23 (57.5)	19 (50)	
Functional status, n (%)			0.898
Independent	31 (77.5)	28 (73.7)	
Mildly dependent	9 (22.5)	10 (26.3)	
Comorbid, n (%)			
Hypertension	25 (62.5)	20 (52.6)	0.38
Diabetes melitus	4 (10)	4 (10.5)	0.94
Osteoarthritis	10 (25)	8 (21.1)	0.68
Osteoporosis	6 (15)	10 (26.3)	0.21
COPD	6 (15)	9 (23.7)	0.33
Cerebrovascular disease	4 (10)	2 (5.3)	0.43
Cognitive function, n (%)			0.246
Normal	35 (87.5)	37 (97.4)	
Moderate cognitive impairment	4 (10)	1 (2.6)	
Severe cognitive impairment	1 (2.5)	0 (0)	
Mental status, n (%)			
Normal	36 (90)	33 (86.8)	0.935
Mild depression	4 (10)	5 (13.2)	
PASE			0.82
Men	34.8 ± 33.82	53.8 ± 38.03	
Women	57 ± 27.28	51.3 ± 31.78	
Laboratorium			
Hb, g/dL	12.52 ± 1.27	12.45 ± 1.43	0.820
Blood sugar, mg/dL	84.50 (46.0–330.0)	86.00 (58.0–408.0)	0.779
Urea, mg/dL	30 (17.0–77.0)	31.0 (15.0–62.0)	0.685
Creatinine, mg/dL	0.79 (0.52–1.77)	0.79 (0.54–1.28)	0.964
eGFR, mL/min/1.73 m^2^	78.35 ± 21.70	79.50 ± 16.64	0.795
Albumin, g/dL	4.35 (3.6–4.9)	4.3 (3.1–4.8)	0.537
IGF-1, ng/dL	77.08 ± 20.9	78.92 ± 21.7	0.705
IL-6, pg/dL	44.51 ± 23.57	36.47 ± 17.45	0.092
Anthropometric			
Body weight (kg), mean ± SD	42.87 ± 9.04	44.75 ± 7.93	0.332
Body Mass Index, mean ± SD	17.62 ± 2.71	17.71 ± 2.72	0.807
Upper arm circumference, mean ± SD	21.74 ± 3.08	22.63 ± 2.87	0.190
Waist circumference, mean ± SD	77.16 ± 10.47	75.62 ± 8.78	0.484
Calf circumference, mean ± SD	27.03 ± 2.40	27.96 ± 2.29	0.083
Intake by food recall			
Calorie (kcal/day)	1428.48 ± 379.82	1605.29 ± 363.63	0.039 *
Men	1478.5 ± 405.91	1818.53 ± 334.1	0.016 *
Women	1398.47 ± 368.53	1450.20 ± 305.61	0.606
Protein (g/day)	52.29 ± 15.72	57.73 ± 16.54	0.140
Men	49.28 ± 14.56	61.64 ± 16.16	0.034 *
Women	54.09 ± 16.39	54.88 ± 16.58	0.870
Carbohydrate (g/day)	188.0 ± 52.37	217 ± 63.78	0.031 *
Men	204.94 ± 46.94	266.57 ± 58.34	0.003 *
Women	177.85 ± 53.71	181.06 ± 38.85	0.818
Fat (g/day)	48.74 ± 21.98	53.13 ± 26.32	0.425
Men	42.41 ± 19.93	55.46 ± 32.2	0.189
Women	52.55 ± 22.66	51.44 ± 21.75	0.866
Zinc (mg/day)	4.32 ± 1.41	5.33 ± 1.93	0.01 *
Men	4.11 ± 1.53	5.27 ± 1.41	0.036 *
Women	4.45 ± 1.35	5.39 ± 2.27	0.088

*: *p*-value < 0.05; ASMI: Appendicular Skeletal Muscle Index; COPD: Chronic Obstructive Pulmonary Disease; eGFR: Estimated Glomerular Filtration Rate; IGF-1: Insulin-like Growth Factor 1; IL-6: Interleukin 6.

**Table 2:** Outcomes measured after the 2-week intervention.

Variable	Intervention Group			Placebo Group			Intervention Effect	
	Baseline	Day 14	*p*-Value	Baseline	Day-14	*p*-Value	Mean Difference (95% CI)	*p*-Value
Calf circumference, cm	27.03 ± 2.4	27.76 ± 2.51	0.02	27.96 ± 2.29	28.16 ± 2.49	0.37	0.53 (−0.22–1.3)	0.16
Men	28.3 ± 1.8	28.7 ± 1.16	0.38	28.7 ± 2.29	29 ± 2.48	0.41	0.1 (−0.96–1.32)	0.74
Women	26.3 ± 2.42	27.2 ± 2.93	0.02	27.4 ± 2.17	27.6 ± 2.38	0.61	0.7 (−0.29–1.79)	0.15
IGF-1, ng/dL	77.08 ± 20.9	81.27 ± 30.22	0.23	78.92 ± 21.7	77.79 ± 27.72	0.8	5.32 (−6.72–17.35)	0.38
Men	82.65 ± 17.35	84.32 ± 29.61	0.42	70.89 ± 24.29	73.28 ± 28.75	0.4	−0.7 (−22.72–24.12)	0.95
Women	73.75 ± 22.43	79.45 ± 31.03	0.23	84.75 ± 17.97	81.06 ± 27.14	0.29	9.38 (−4.25–23.01)	0.17
IL-6, pg/dL	44.51 ± 23.57	33.41 ± 17.83	<0.001 *	36.47 ± 17.45	61.39 ± 35.26	0.00 *	−36 (−49.2–(−22.67))	<0.001 *
Men	42.31 ± 22.09	27.98 ± 13.38	0.02 *	41.06 ± 21.29	62.53 ± 28.04	0.01 *	−35.79 (−53.73–(−17.85))	0.00 *
Women	45.83 ± 24.76	36.67 ± 19.56	0.07	33.14 ± 13.6	60.56 ± 40.34	0.002 *	−36.58 (−55.6–(−17.55))	0.00 *
ASMI, kg/m^2^								
Men	6.49 ± 0.39	6.56 ± 0.56	0.36	6.43 ± 0.46	6.58 ± 0.55	0.14	−0.08 (−0.39–0.23)	0.62
Women	5.43 ± 0.28	5.58 ± 0.42	0.03 *	5.48 ± 0.2	5.59 ± 0.29	0.03 *	0.04 (−0.12–0.21)	0.62
Handgrip strength, kg								
Men	22.2 ± 5.48	23.2 ± 5.89	0.12	23.44 ± 6.62	25 ± 7.21	0.03 *	−0.56 (−2.41–1.28)	0.54
Women	15.88 ± 5.54	17.48 ± 5.11	<0.001 *	16.5 ± 4.5	17.23 ± 4.58	0.18	0.87 (−0.64–2.38)	0.25
Gait speed, m/s								
Men	0.72 ± 0.22	0.79 ± 0.21	0.02 *	0.71 ± 0.22	0.77 ± 0.25	0.05	0.21 (−0.06–0.1)	0.61
Women	0.69 ± 0.2	0.73 ± 0.26	0.16	0.68 ± 0.22	0.77 ± 0.25	0.11	−0.05 (−0.13–0.08)	0.64
Creatinine, mg/dL	0.87 ± 0.28	0.89 ± 0.34	0.41	0.83 ± 0.2	0.86 ± 0.26	0.39	0.01 (−0.08–0.07)	0.93

*: *p*-value < 0.05; ASMI: Appendicular Skeletal Muscle Index; IGF-1: Insulin-like Growth Factor 1; IL-6: Interleukin 6.

## Data Availability

The data presented in this study are available in this article.
